# Effectiveness of hormones in postmenopausal pelvic floor dysfunction—International Urogynecological Association research and development—committee opinion

**DOI:** 10.1007/s00192-019-04070-0

**Published:** 2019-08-07

**Authors:** Barbara Bodner-Adler, May Alarab, Alejandra M. Ruiz-Zapata, Pallavi Latthe

**Affiliations:** 1 Department of General Gynecology and Gynecologic Oncology, Medical University of Vienna, Währinger Gürtel 18-20, 1090 Vienna, Austria; 2 Division of Urogynecology and Reconstructive Pelvic Surgery, Department of OBS/GYN, Mount Sinai Hospital, University of Toronto, Toronto, Ontario Canada; 3 Department of Obstetrics and Gynecology, Department of Urology, Radboud Institute for Molecular Life Science (RIMLS) Radboud University Medical Center, Nijmegen, The Netherlands; 4 Department of Urogynecology, Birmingham Women’s and Children’s NHS Foundation Trust, Edgbaston, UK

**Keywords:** Urinary incontinence, Pelvic organ prolapse, Vulvovaginal symptoms, Sex steroids, Hormonal treatment, Postmenopausal women

## Abstract

**Introduction and hypothesis:**

There is clear evidence of the presence of estradiol receptors (ERs) in the female lower urinary and genital tract. Furthermore, it is a fact that estrogen deficiency after menopause may cause atrophic changes of the urogenital tract as well as various urinary symptoms. Moreover, the effect of hormone replacement therapy (HRT) on urinary incontinence (UI) symptoms as well as pelvic organ prolapse (POP), anal incontinence (AI) and vulvovaginal symptoms (VVS) is still a matter of debate. This committee opinion paper summarizes the best evidence on influence of sex steroids as well as hormonal treatment (local and systemic) in postmenopausal women with pelvic floor disorders.

**Methods:**

A working subcommittee from the International Urogynecology Association (IUGA) Research and Development Committee was formed. A thorough literature search was conducted and an opinion statement expressed. The literature regarding hormones and pelvic floor disorders was reviewed independently and summarized by the individual members of the sub-committee.

**Results:**

The majority of studies reported that vaginal estrogen treatment when compared with placebo has more beneficial effects on symptoms and signs of vaginal atrophy including sensation of burning, dyspareunia and UI symptoms. Definitive evidence on local estrogen application and prolapse treatment or prevention is lacking. A statistically significant increase in risk of worsening of UI as well as development of de novo incontinence was observed with estrogen-only or combination systemic HRT.

**Conclusions:**

In summary, local estrogen seems to be safe and effective in the treatment of VVS and can also improve urinary symptoms in postmenopausal patients with UI, but most of these recommendations correspond to evidence level 2C. The evidence in POP is still scarce but not in favor of benefit. Finally, the duration of local estrogen treatment (LET), optimal dosage, long-term effects and cost-effectiveness compared with current practice are still unknown.

## Introduction

Pelvic floor disorders (PFD) are common gynecologic complaints that adversely affect the quality of life (QoL) of many postmenopausal women because of their impact on physical, social, emotional and sexual wellbeing [1]. Although it is difficult to separate the effects of declining estrogen levels in menopause from aging in general, it is clear that the pelvic organs and their surrounding muscular and connective tissue support are estrogen-responsive [2]. Epidemiologic studies indicate that menopause is a major risk factor for the development of PFD, and severity of these disorders increases significantly after menopause [3].

Over the last few years there has also been much discussion and controversy regarding local and systemic hormone replacement in postmenopausal women with PFD. Even though large data sets and clinical trials on the effect of systemic HRT exist [4, 5], the effect of hormonal therapy (HT) in menopause on PFD is still a matter of debate.

The aim of this review was to look at the evidence on the role of hormones in pelvic floor disorders. A review of the literature was performed to summarize the evidence on influence of sex steroids as well as local and systemic HRT in postmenopausal women with UI, urinary tract infection (UTI), VVS, POP and AI.

## Methods

This opinion paper focuses on the effectiveness of hormones in PFDs. A thorough search of the literature was conducted independently by the four members of the sub-committee (BBA, MA, AR, PL). Our keywords comprised *pelvic floor dysfunction* AND *disorders* AND *hormones* AND *estrogen* AND * treatment* AND *therapy* AND *postmenopausal women* for PubMed. The findings of all relevant studies were summarized. The limits for literature search were adult females. We used no language restriction for screening.

## Role of estrogens and estrogen receptors in the female lower urinary and genital tract

### Role of endogenous estrogens

Estrogens are vasoactive hormones; therefore, they can increase blood flow, which helps maintain the low pH (by increasing glycogen, which increases lactobacilli, which lowers pH) in the vagina necessary to protect it against UTIs and vaginitis [6]. They can also influence the central neurologic control of micturition and improve the maturation index of the urethral squamous epithelium [7]. They can affect detrusor function through modifications in muscarinic receptors and by inhibition of the extracellular calcium ions into the muscle cells [7]. In menopause, not only is there a deficit in estrogen production, but also a downregulation of estrogen receptor (ER) β and progesterone receptor (PR) in the vaginal wall of postmenopausal women [8].

### Role of estrogen receptors

ERs are found in the female squamous epithelium of the proximal and distal urethra, vagina and trigone of the bladder and in the squamous epithelium of the anal canal [7]. Furthermore, they are expressed in the para-urethral tissues, urethral sphincter, uterosacral ligaments and pelvic floor musculature [8].

## Role of local estrogens in the treatment of pelvic floor disorders

### Local estrogen in the treatment of lower urinary tract symptoms (LUTS)

A systematic review of 44 individual clinical studies from 2014 concluded that all available vaginal estrogen preparations reduce LUTS, including urinary urgency, frequency, lower urinary tract pain, urgency urinary incontinence and voiding dysfunction [9]. Furthermore, some studies reported that local estrogen treatment (LET) improved the overall, subjective, objective and urodynamic variables in UI and overactive bladder (OAB); also the mid-urethral closure pressure (MUCP) seemed greatest in patients treated with estrogens compared with pelvic floor exercises or electro-stimulation [10].

### Local estrogen in the treatment of OAB syndrome

In OAB, the use of LET has demonstrated efficacy in clinical trials. Randomized, placebo-controlled trials and also various guidelines have demonstrated significant improvement over placebo in urinary frequency, nocturia, urgency incontinence and volume at first sensation to void [11, 12]. Moreover, Erikson et al. performed a double-blind, randomized, placebo-controlled trial which showed that frequency, urgency, urgency incontinence and stress incontinence significantly improved with LET. However, one has to mention that some of the subjective improvement in these symptoms may simply represent local estrogenic effects reversing urogenital atrophy rather than a direct effect on lower urinary tract function [13]. In an RCT published by Nelken et al., postmenopausal patients with OAB symptoms received either an estradiol-releasing vaginal ring or oral oxybutynin 5 mg twice daily. Those women who received oral oxybutynin had a mean decrease of 3.0 voids per day compared with a decrease of 4.5 voids per day in women using the estradiol ring, with a significant improvement in QoL parameters in both groups [14]. The authors concluded that the estradiol-releasing vaginal ring and oral oxybutynin are similarly effective in OAB syndrome.

Regarding the synergistic use of vaginal estrogen therapy with antimuscarinic therapy, a 12-week prospective randomized trial compared tolterodine 2 mg twice daily and vaginal conjugated estrogen cream versus tolterodine 2 mg twice daily alone in 80 postmenopausal women with OAB symptoms. The results showed that combination therapy had a significantly greater improvement in mean daytime frequency and voided volume than monotherapy [15]. Furthermore, a significantly greater improvement in quality of life (QoL) parameters was also observed in the combination therapy group.

Contrarily, other RCTs demonstrated that vaginal estrogen showed equivalence to oxybutynin 5 mg in the treatment of urinary urgency, frequency, and urge urinary incontinence, although the oral anticholinergic demonstrated a higher rate of side effects [14, 16, 17]. The addition of local estrogen (various methods) to tolterodine (immediate or extended-release forms) did not offer an advantage over tolterodine alone, but one of the studies did demonstrate significantly fewer voids per day and greater voided volumes in the tolterodine plus estrogen arm [15].

### Selective estrogen receptor modulators (SERMs) in the treatment of OAB

Regarding SERMs and OAB, Schiavi et al. assessed the effectiveness of Ospemifene in the improvement of the urgency component in women with MUI who underwent mid-urethral sling (MUS) surgery. After surgical intervention, 38/81 (47%) patients received Ospemifene (60 mg/day for 3 months), and a significant difference was observed regarding mean number of voids, urgent micturition episodes/24 h, UUI, nocturia events and OAB-QoL symptoms. The authors concluded that Ospemifene is an effective therapy after MUSs in women with MUI, improving urgency symptoms and quality of life [18]. Another study, also published by Schiavi et al., demonstrated that Ospemifene is also an effective therapy for postmenopausal women with VVA affected by OAB, improving sexual function and quality of life [19].

### Local estrogen in the treatment of VVS

In postmenopausal women with vaginal dryness, itching, pain or burning, meta-analyses of randomized controlled trials (RCTs) concluded that vaginal estrogen cream use reduced symptoms in the majority of women, but only few women used them beyond 6 months [20]. A systematic review, published in 2014, concluded that all commercially available vaginal estrogens effectively relieve common vulvovaginal atrophy-related complaints and have additional utility in patients with urinary urgency, frequency or nocturia, stress urinary incontinence (SUI) and UUI, and recurrent UTIs [2]. However, a recent large RCT concluded that neither the vaginal estradiol tablet nor the over-the-counter vaginal moisturizer provided additional benefit over placebo in reducing postmenopausal VVS [21].

### Local estrogen in the treatment of recurrent UTI (rUTI)

It is well known that menopause brings a reduction in vaginal estrogen, an increase in vaginal pH and alteration in the vaginal microbiota away from the lactobacillus-dominant environment, resulting in an increase of recurrent UTI. A recent rapid review, published by Smith et al. in 2018, summarized five RCTs (overall sample size 444) on the use of local estrogen and recurrent UTI. Meta- analyses showed that vaginal estrogen prevents rUTI in postmenopausal women [22].

### Local estrogen in the treatment of POP

Regarding POP, the role of LET is unclear. The extracellular matrix (ECM) is a key constituent of the supportive tissue of the vagina, and alterations in ECM metabolism have been demonstrated in women with POP [23]. Other studies have shown that matrix metalloproteinases (MMPs) synthesis and activity were suppressed in the presence of estrogen, leading to decreased collagen degradation in pelvic floor connective tissue [2]. Although estrogen supplementation has not been established as an effective preventive or therapeutic measure for POP, vaginal estrogen is often used to reduce side effects associated with conservative treatments (such as pessaries) and surgically implanted materials [24]. The only prospective study of 120 postmenopausal women, using pessaries with and without vaginal estrogen, found a trend towards a higher rate of complications among non-users of estrogen [25]. Dessie et al. conducted a retrospective cohort study of 134 women treated with pessaries for at least 3 months. Their results demonstrated that women using vaginal estrogen were less likely to discontinue their pessary (30.6% vs. 58.5%, *P* < 0.001) or develop vaginal discharge [hazard ratio (HR) 0.31, 95% CI 0.17–0.58], but it was not protective against erosion (HR 0.93, 95% CI 0.54–1.6) or vaginal bleeding (HR 0.78, 95% CI 0.36–1.7) [26].

Furthermore, in an RCT by Karp et al., using an estradiol-releasing ring 2 weeks (for 12 weeks) after pelvic floor repair resulted in improved markers of tissue quality including slightly reduced urinary tract infections compared with placebo [27]. In a study evaluating the role of 2–12 weeks’ preoperative local estrogen in increasing vaginal wall thickness prior to POP surgery, there was no statistically significant increase in the thickness of the vagina in the treatment group compared with the group with no intervention [28]. Similar, Rahn et al. summarized in an SR that it is uncertain whether preoperative vaginal estrogen is beneficial before prolapse repair as no increased vaginal subepithelial or muscularis thickness could be observed (low quality) [16]. Furthermore, preoperative vaginal estrogen decreased the frequency of bacteriuria in the first postoperative month, but no difference was seen for symptomatic cystitis (very low quality) [16].

### Local estrogen in the treatment of fecal incontinence

Regarding AI, one small RCT compared local estrogen with placebo in 36 menopausal women with fecal incontinence (FI). The authors found no difference between the groups with respect to subjective improvement assessed by the Wexner score [29].

Table 1 summarizes the study quality in general as well as the profile of evidence regarding pelvic floor disorders and efficacy of LET.

**Table 1:** Efficacy of local estrogen therapy (vaginal estrogen versus placebo or no treatment) on pelvic floor disorders: quality of studies and profile of evidence

PFD	Quality of studies^a^	Parameters	Outcomes	Qualityof evidence^b^
VA	Fair	Subj. +obj.	Improved	Moderate
OAB	Fair	Subjective	Improved	Low
SUI	Fair	Subjective	Improved	Low + moderate
UI	Fair	Objective	Improved	Low-moderate
POP	Poor	Subjective	Unclear	Low

^a^Quality of studies: good-fair-poor (Agency for Healthcare Research and Quality). Owens DK et al. (2010) AHRQ series paper 5: grading the strength of a body of evidence when comparing medical interventions–Agency for Healthcare Research and Quality and the effective health-care program. J Clin Epidemiol. 63: 513–23

^b^Grade of evidence: high-moderate-poor (Grades for Recommendation, Assessment, Development and Evaluation system). Atkins D et al. (2004) Systems for grading the quality of evidence and the strength of recommendations I: critical appraisal of existing approaches. The GRADE Working Group. BMC Health Serv Res. 4: 38

Abbreviations

*PFD* pelvic floor disorders, *VA* vaginal atrophy, *Subj. + obj.* subjective + objective, *UI* urinary incontinence, *OAB symptoms* overactive bladder symptoms, *SUI* stress urinary incontinence, *POP* pelvic organ prolapse

## Role of systemic hormonal treatment relating to pelvic floor disorders

### Influence of HRT on UI

There are major discrepancies regarding the role of exogenous systemic estrogen administration for UI in the existing literature, and available clinical trials report conflicting results. On the one hand, subjective improvement of UI could be demonstrated in a few studies, but several clinical trials and reviews could not demonstrate any efficacy—quite the contrary—of estrogen therapy for treatment of UI [30–32]. However, these results have to be interpreted with caution as different therapeutic interventions were used in terms of the type of estrogen, dose, route of administration and duration of therapy.

Before publication of the Women’s Health Initiative (WHI) in 2002, systemic hormone therapy (HT) in various forms, doses and regimens of estrogen with or without progestin was commonly prescribed [4]. Outcomes from the WHI study also taught us that systemic estrogen can be harmful because of the reported side effects such as increased risk of stroke after some years. A subgroup analysis showed that women receiving estrogen only as well as combination therapy had a statistically significant increase in risk for UI [estimated event rate difference, per 10,000 person-years (95% CI) 1261 (880–1689) and 876 (606–1168)], but this did not persist after stopping hormone therapy [4]. Cody et al. reported in a Cochrane review (the combined results of six trials) that administration of oral estrogens resulted in an impairment of UI compared with placebo [risk ratio (RR) 1.32, 95% CI 1.17–1.48]. All of the women were hysterectomized, and the treatment used was conjugated equine estrogen. The results for women with an intact uterus where estrogen and progestogen were combined also showed a statistically significant worsening of UI (RR 1.11, 95% CI 1.04–1.18) [30]. Furthermore, women who were continent and received systemic estrogen replacement, with or without progestogens, for reasons other than UI, are more likely to report the development of new UI [30]. In summary, systemic HT should not be used either to prevent or as therapy for urinary incontinence.

### Influence of HRT on FI

Staller et al. studied the association between HRT and risk of FI among 55.828 postmenopausal women (participating in the WHI, Nurses’ Health Study as well as HERS), and current or past use of HRT was associated with a modestly increased risk of FI among postmenopausal women. These results support a potential role for exogenous estrogens in the impairment of the fecal continence mechanism [4, 5, 33].

## Side effects of estrogen treatment

No significant adverse events were reported in cases treated with topical estrogen [10]. A Cochrane review, dealing with topical estrogen as well as with systemic estrogen administration, reported common side effects such as breakthrough bleeding, breast tenderness or nausea in some cases [30].

## Discussion

The relationship between hormones and pelvic floor disorders is still not entirely clear. Vaginal estrogens may play a useful role as an adjunct in the management of common PFDs in postmenopausal women, and oral systemic estrogen administration seems to worsen UI [2, 10, 30].

Regarding the effect of local estrogen on prolapse symptoms, evidence is scarce, or absent. A Cochrane review by Ismail et al. did not find any clear evidence to suggest whether estrogens help in reducing the symptoms and concluded that an adequately powered RCT with long-term follow-up is needed to identify the benefits or risks associated with estrogen supplementation in the prevention and management of POP [34].

Regarding the effect of local estrogen on OAB symptoms, the use of local estrogen seems to be of clinical importance. Postmenopausal women are frequently treated with anti-cholinergic drugs, but the side effects cause many women to discontinue the medication. As one study demonstrated equivalent improvements with an ultra-low dose vaginal estradiol ring compared with an immediate-release oral anticholinergic, the use of an estradiol ring provides another treatment option to avoid this common malady. Nevertheless, it is currently unknown whether other anticholinergic drugs will be superior to vaginal estrogens alone in the management of OAB.

## Limitation of the literature

A wide variety of topical estrogen administration forms and different dosages were used in the reported studies. Furthermore, heterogeneous control groups were used, and LET was compared with placebo or no treatment or non-hormonal treatment. There were a few RCTs, but they were not adequately powered. Therefore, most of the results have to be interpreted with caution.

### Summary and recommendations

Most of the recommendations are evidence level 2C, meaning a low quality of evidence, and there is still a need for additional robust evidence.

- LET seems to be safe and beneficial in the treatment of VVS compared with placebo.
- LET improves lower urinary tract symptoms such as frequency, urgency and UI in postmenopausal women.
- LET improves the vaginal environment and reduces UTIs at the time of prolapse surgery.
- Little evidence exists to support the effect of LET on the quality of surgical repair, prolapse recurrence, etc.
- Postmenopausal women on systemic hormonal treatment should be counseled regarding the increased risk of worsening of urinary incontinence symptoms or development of de novo incontinence and possibly fecal incontinence.

Still unclear:

- Evidence is too sparse to give any recommendations regarding the effectiveness of LET in the treatment or prevention for POP.
- No evidence and recommendations exist regarding the duration, optimal dosage, long-term effect and cost-effectiveness of estrogen treatment.
